# IL-8–NF-κB–ALDH1A1 loop promotes the progression of intrahepatic cholangiocarcinoma

**DOI:** 10.1097/HC9.0000000000000664

**Published:** 2025-02-26

**Authors:** Yinghui Song, Yufeng Li, Jia Zhou, Jianfeng Yu, Qianwei Hu, Feicheng Yang, Zexi Yin, Yizhi Wang, Yueren Wang, Xinling Zhang, Yuewei Tao, Chuang Peng, Sulai Liu

**Affiliations:** 1 Central Laboratory, Hunan Provincial People’s Hospital/The First Affiliated Hospital of Hunan Normal University, Changsha, Hunan, P. R. China; 2 Department of Hepatobiliary Surgery, Hunan Provincial People’s Hospital/The First Affiliated Hospital of Hunan Normal University, Changsha, Hunan, P. R. China; 3 Department of Pathology, Hunan Provincial People’s Hospital/The First Affiliated Hospital of Hunan Normal University, Changsha, Hunan, P. R. China; 4 Department of Pediatrics, Hunan Provincial People’s Hospital/The First Affiliated Hospital of Hunan Normal University, Changsha, Hunan, P. R. China; 5 School of Medicine, University of Dundee, Ninewells Hospital, Dundee, UK

**Keywords:** ALDH1A1, IL-8/CXCR2, intrahepatic cholangiocarcinoma, NF-κB

## Abstract

**Background:**

Intrahepatic cholangiocarcinoma (ICC) is a poor prognosis of malignant cancer with high lymph node metastasis and resistance to systemic therapies. Recent studies suggested that the involvement of IL-8 could promote ICC metastasis through epithelial–mesenchymal transition while the ICC-ALDH1A1^high^ subtype is clarified by multi-omics study. The correlation between ALDH1A1 and IL-8 in ICC remains elusive. This study aims to further explore the roles and regulatory mechanisms of ALDH1A1 and IL-8 in ICC.

**Methods:**

We analyzed IL-8 and ALDH1A1 expression in ICC patients and cells. CXCR2 inhibitor (SB225002) was applied to inhibit the function of IL-8, and JSH-23 was applied to inhibit the NF-κB signaling pathway. We examined the effects of IL-8 inhibition on NF-κB, ALDH1A1 expression, and cell growth, migration, invasion, and stemness. Moreover, we examined the effects of ALDH1A1 on NF-κB, IL-8 expression, and cell growth, migration, invasion, and stemness. The effects of IL-8 and ALDH1A1 on tumor growth and NF-κB expression were validated using subcutaneous tumors in nude mice.

**Results:**

IL-8-derived tumor cells could promote ICC progression. The high expression of IL-8 in serum was associated with lymph node metastasis. IL-8 could upregulate ALDH1A1 expression by activating the NF-κB signaling pathway, promoting tumor progression. Upregulation of ALDH1A1 could activate NF-κB to promote IL-8 secretion, forming a positive feedback loop to promote tumor invasiveness and cell stemness in ICC.

**Conclusions:**

IL-8-derived tumor cells could upregulate ALDH1A1 expression by activating the NF-κB signaling pathway, promoting tumor progression. Upregulation of ALDH1A1 could activate NF-κB to promote IL-8 secretion, forming a positive feedback loop to promote tumor invasiveness and cell stemness in ICC.

## INTRODUCTION

Intrahepatic cholangiocarcinoma (ICC) refers to a malignant tumor originating from the epithelium of the intrahepatic bile duct, slightly larger in males than females. In recent years, the incidence rate of ICC has increased significantly. At present, the etiology of ICC is not fully understood and may be related to intrahepatic bile duct stones, viral hepatitis, cirrhosis, primary sclerosing cholangitis, congenital biliary malformations, etc. In addition, parasitic infection, exposure to toxic and harmful substances, and abnormal metabolism (diabetes and obesity) are also considered risk factors for ICC.^1^ ICC has no obvious symptoms in the early stages and is mostly diagnosed in the middle and late stages. Currently, surgery is the preferred treatment for ICC. Due to the high risk of lymph node metastasis in ICC, some patients still experience recurrence and metastasis after radical surgical resection. With the advancement of immunotherapy, PD-1 antibodies have been used to attempt treatment of ICC, but only a very small fraction of patients benefit.^2^ Therefore, it is meaningful to elucidate the biological behavior of ICC and provide new ideas for finding treatment strategies for ICC.

IL-8 is a cytokine in the chemokine family that binds to its specific receptor and exerts its effects. Previous studies have shown that IL-8 is mainly derived from non-tumor cells and exerts its pro-tumor effect indirectly.^3^ There are also studies indicating that IL-8 secreted by tumor cells also plays a role in promoting tumor growth.^4^ The level of IL-8 secreted by tumors is closely related to the efficacy of immunotherapy. Therefore IL-8 is attracting increasing attention in tumor research.^5^ IL-8 secreted by macrophages or bile duct epithelial cells stimulated by high amylase bile promotes the occurrence of ICC.^6^ Moreover, it is reported that IL-8–CXCR2–PI3K/AKT axis regulates CD97 expression and promotes ICC metastasis through epithelial–mesenchymal transition.^7^ Exploring the function of IL-8 may provide new insights for the treatment of ICC. ALDH1A1, as a stem cell marker molecule, has been confirmed to be upregulated in various tumors.^8^ The study of precise stratification of ICC based on multi-omics revealed an important precise molecular subtype in the ICC-ALDH1A1^high^ subtype.^9^ All this evidence suggests it is necessary to explore the mechanism of ALDH1A1 in ICC.

Previous studies have shown that in triple-negative breast cancer, paclitaxel can promote stem cell phenotype by inducing the secretion of IL-8 while inhibiting the activation of NF-κB can reduce the production of cancer stem cells (CSCs).^10^ Stimulating breast cancer cell MDA-MB-231 with IL-8 can increase ALDH^positive^ cells, while CXCR2-specific inhibitor SB225002 can inhibit this effect of IL-8. In addition, ALDH^positive^ cells exhibit higher levels of IL-8.^11^ ALDH1A1 can also promote tumor angiogenesis in melanoma by activating the IL-8/Notch signaling pathway.^12^ However, there have been no reports on the correlation between ALDH1A1 and IL-8 in ICC. This study aims to further explore the roles and regulatory mechanisms of ALDH1A1 and IL-8 in ICC, providing a theoretical basis for deepening the understanding of the biological behavior of ICC and providing a scientific basis for further effect targeted therapy of ICC.

## METHODS

### Patient characteristics

Thirty patients were enrolled following approval from the ethical committee and informed consent. The enrolled patients were between 42 and 68 years old, diagnosed with ICC confirmed by histopathologic evaluation, and received surgery resection at Hunan People’s Hospital in 2023. Peripheral blood samples (2 mL) from the patients with ICC before and after surgery were obtained and collected in anticoagulant sterile tubes for ELISA assay. For comparison, 30 healthy donors were evaluated as normal controls. The tumor tissues and adjacent tissues of patients with ICC were also collected for western blot and immunohistochemistry (IHC) assay. The public data was obtained from the TCGA database (https://portal.gdc.cancer.gov). The data was organized using the STAR process. The RNAseq data was extracted by TPM format data. Next, the log2 (value+1) method was used to process and analyze the data.

### Cell culture and lentiviral infection

All the cells in this study were purchased from ATCC or a national collection of authenticated cell cultures. Cells were cultured in RPMI 1640 or DMEM medium with 10% fetal bovine serum (FBS) and 100 U/mL penicillin/streptomycin (Gibco). Cultures were incubated at 37°C according to the instructions. To generate stable si-ALDH1A1/ov-ALDH1A1 expressing cell lines, lentiviruses were used to infect HuCCT1 cells with polybrene supplementation (10 mg/mL). Next, puromycin (2 mg/mL) in a complete medium was used for selection (Genechem).

### CCK-8 assay

The logarithmic growth phase cells were inoculated into a 96-well plate at 5000 cells per well. After the cells adhered to the wall, they were treated accordingly. Then, the cells were cultured for 24 hours, 48 hours, or 72 hours. Next, 10 μL CCK-8 reagent (Dojindo) was added to each well, incubating at 37°C for 1 hour. The absorbance value at 450 nm of each well was recorded.

### ELISA

The serum or cell culture supernatant was collected, and a 100 μL detection sample was added to each well. The production of IL-8 or ALDH1A1 was detected according to the instructions of the ELISA detection kit (Jonlnbio).

### Colony formation assay

ICC cells were treated accordingly and prepared as a single-cell suspension. Next, 2 mL per well of culture medium was added to a 6-well plate at a density of 500 cells. The cells were cultured for another 14 days and stained with Giemsa. The colons were counted manually after being fixed with absolute ethanol. Colonies containing more than 50 cells were considered survivors.

### Flow cytometry

Apoptosis was measured using the Annexin V/fluorescein isothiocyanate method according to the manufacturer’s instructions (Multisciences). Briefly, ICC cells were treated accordingly and collected using 0.25% trypsin, washed with cold PBS twice, resuspended in 300 μL of binding buffer, and stained with 5 μL of fluorescein isothiocyanate-labeled Annexin V and 5 μL of propidium iodide. After 15 minutes of incubation, 200 μL of binding buffer was added before analysis. The apoptosis ratio was then analyzed using CytoFLEX analysis software (Beckman Coulter Life Sciences) on CytoFLEX flow cytometer (Beckman Coulter). ICC cells were treated with IL-8, IL-8 combined with SB225002, or IL-8 combined with JSH-23 for 24 hours; then cells were harvested and stained with 0.4 μg anti-human CD133 and rabbit IgG isotype control, phycoerythrin-conjugated. The fluorescence intensity was analyzed using CytoFLEX analysis software (Beckman Coulter Life Sciences) on CytoFLEX flow cytometer (Beckman Coulter).

### Wound-healing assay

Cells were seeded into 6-well plates at a density of 5×10^6^ cells per well with 2 mL of medium. After 24 hours, the medium was removed. A micropipette tip was then used to scratch the surface of adherent ICC cells along the central axis of the 6-well plate. Next, a drug-containing 1640 medium without FBS was added to the 6-well plate, and the cells were cultured for 48 hours. Then, the pictures were taken under a microscope. ImageJ software was applied to analyze the scratch healing area, and the 48-hour cell scratch healing rate was calculated. Scratch healing rate (%)=(initial scratch width 48-hour scratch width)/initial scratch width 100.

### Transwell chamber experiment

Cells were seeded into 6-well plates at a density of 5×10^6^ cells per well with 2 mL of medium. After 24 hours, a drug-containing 1640 medium without FBS was added to the 6-well plate, and the cells were cultured for 24 hours. The cells were then trypsinized, and 2.5×10^4^ cells were seeded in the upper chamber of Transwell. Meanwhile, 500 μL of complete medium containing 10% FBS was added to the lower chamber. The cells were cultured for another 48 hours. After that, the chamber was taken out and washed with PBS. The cells were then fixed with 4% paraformaldehyde for 15 minutes, washed 3 times with PBS, stained with 0.1% crystal violet for 10 minutes, and washed 3 times with PBS. Then, the pictures were selected randomly under a microscope. The number of migrated cells was counted.

### Quantitative reverse transcription polymerase chain reaction

A quantitative reverse transcription polymerase chain reaction was performed as described. Briefly, the cells were harvested, and the total RNA was extracted using TRIzol reagent (Invitrogen). The total RNA (1 μg) was used for cDNA synthesis. The RevertAid RT reverse transcription kit (Thermo Fisher Scientific) was applied to perform reverse transcription of RNA, and then quantitative reverse transcription polymerase chain reaction was performed on the resulting cDNA using a SYBR-Green fluorescence-based assay kit (Applied Biosystems) and an ABI Prism 7500 sequence detection system (Applied Biosystems). The glyceraldehyde-3-phosphate dehydrogenase amplification level was used as an endogenous control to normalize the amplification of the target gene. The detailed primes sequences information was provided in Supplemental Table S1, http://links.lww.com/HC9/B914.

### Western blot

A western blot experiment was performed as previously described.^4^ The whole cell protein was extracted by using cell lysis buffer. The protein concentration was determined using the method described in the Micro BCA protein determination kit (Thermo Fisher Scientific). The total protein (20 μg) was loaded into each well. The detailed antibody information was provided in Supplemental Table S2, http://links.lww.com/HC9/B914.

### Immunohistochemistry

For the IHC study, tumor tissues were stored in 4% paraformaldehyde for more than 24 hours and cut into 4-mm-thick slices. The paraffin wax was then removed and stained with IHC using a method described previously.^13^

### Cell immunofluorescence staining

Cells were fixed with 4% formaldehyde at room temperature for 30 minutes. After fixation, the slide was washed 3 times with PBS, 0.1% TritonX-100 was added at 100 μL/slide, and then incubated at room temperature for 10 minutes. Next, the slide was washed 3 times with PBS, and 10% FBS PBS was added at 100 μL/slide for blocking. The dilution of the primary antibody was added at 100 μL/slide and incubated at room temperature for 1 hour. Next, the slide was washed 3 times with 1×PBS. The secondary antibody was added at 100 μL/slide and incubated at room temperature for 1 hour. Next, the slide was washed 3 times with PBS. The DAPI staining was added at 100 μL/slide and incubated at room temperature for 5 minutes. After the nuclear staining was completed, the slide was washed 3 times with 1×PBS, then washed 3 times with ultrapure water. The anti-quenching sealing tablets were dripped onto glass slides. The slide was dried and stored at −20°C in the dark.

### Tumorsphere formation assay

ICC cells were harvested and prepared as a single-cell suspension (1×10^3^ cells). The cells were seeded into a 6-well ultralow attachment plate (Corning Glass) and were incubated in DMEM deprived of serum comprising 20 ng/mL EGF, 20 ng/mL human fibroblast growth factor basic as well as 20 μL/mL B27 (Peprotech). Under a light microscope (Thermo Fisher Scientific), the formed spheres were calculated 10 days later.

### Tumor xenograft assay

ICC cells with 5×10^6^ cells in 100 μL were s.c. injected into the upper flank of 4–5-week-old nude mice (Slack Jingda Experimental Animal Co., Ltd). The mice in the IL-8 treatment group were i.p. injected with IL-8 at 0.05 mg/kg twice per week. The mice in the SB225002 treatment group were i.p. injected with SB225002 at 10 mg/kg twice per week. The size of the tumor xenograft was checked every 3 days. The tumor xenografts were resected and weighed. The use and care of the mice for this study were reviewed and approved by the Institutional Animal Committee of Hunan Provincial People’s Hospital (SYXK-Xiang-2020-0017).

### Statistical analysis

Statistical analyses were performed using SPSS IBM 20.0. Statistical significance was determined by the *t* test or ANOVA and the Mann–Whitney test (*p*<0.05). GraphPad Prim 8 was applied to generate the figures.

## RESULTS

### IL-8 promotes ICC progression

**FIGURE 1:**
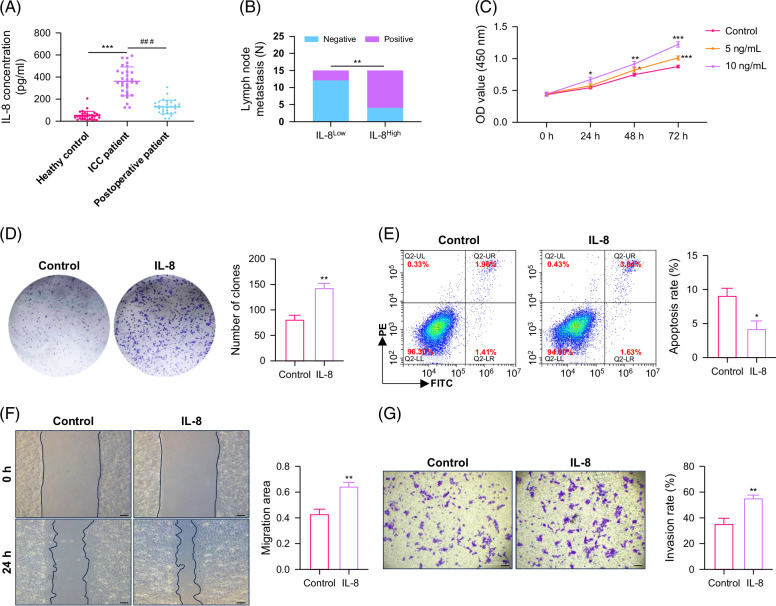
IL-8 promotes ICC progression. (A) The level of IL-8 concentration in 30 patients with ICC. (B) The lymph node metastasis of different IL-8 expression. (C) The proliferation ability of HuCCT1 cells with IL-8 treatment. (D) The clone formation ability of HuCCT1 cells with 10 ng/mL IL-8 treatment. (E) The apoptosis ratio of HuCCT1 cells with 10 ng/mL IL-8 treatment. (F) The migration ability of HuCCT1 cells with 10 ng/mL IL-8 treatment (scale bar=200 μm). (G) The invasion ability of HuCCT1 cells with 10 ng/mL IL-8 treatment (scale bar=200 μm). *p<0.05, **p<0.01, and ***p<0.001. Abbreviations: FITC, fluorescein isothiocyanate; ICC, intrahepatic cholangiocarcinoma; PE, phycoerythrin.

IL-8 was upregulated in various tumors, including cholangiocarcinoma based on the TCGA database analysis, indicating that IL-8 may be associated with the occurrence and development of tumors (Supplemental Figure S1A, http://links.lww.com/HC9/B915). Previously, it was found that IL-8 can come from multiple sources, including tumor cells, endothelial cells, immune cells, etc.^3^,^4^ Subsequently, we detected the levels of IL-8 in various digestive system cell lines. It was found that varying degrees of IL-8 elevation in tumor cells, which is consistent with the findings in the TCGA database (Supplemental Figures S1B and C, http://links.lww.com/HC9/B915). We ultimately focused on ICC cells and found that HCCC-9810, HUCCT1, and RBE all had relatively high levels of IL-8 expression and secretion. Another study showed that IL-8 was highly expressed in 68.8% (86/125) of patients with cholangiocarcinoma, as determined by IHC analysis.^7^ We speculate that IL-8 is likely derived from tumor cells in ICC. Subsequently, we found that patients with ICC had higher levels of IL-8 expression compared to healthy controls by detecting the expression of IL-8 in the serum of ICC patients. Moreover, the expression level of IL-8 was significantly reduced after surgical resection compared to those before surgery, indicating a correlation between tumor burden and IL-8 levels. IL-8 may mainly come from the tumor (Figure 1A).

Furthermore, 30 patients with ICC were divided into a low IL-8 expression group and a high IL-8 expression group based on the median expression level of IL-8. The clinical characteristics of these patients were analyzed. It was found that those with high IL-8 expression had more lymph node metastasis (Figure 1B). Specifically, there were 11 cases of lymph node metastasis that occurred in the IL-8 high expression group, while only 3 cases occurred in the low expression group, indicating a close correlation between IL-8 expression and lymph node metastasis (*p*=0.009). The other information on 30 patients with ICC was provided in Supplemental Table S3, http://links.lww.com/HC9/B914. Subsequently, the exogenous IL-8 was added to tumor cells, and the results showed that IL-8 could promote tumor cell proliferation (Figure 1C). Treating tumor cells with 10 ng/mL IL-8 could significantly promote tumor cell colony formation, inhibit tumor cell apoptosis, and promote tumor cell migration and invasion (Figures 1D–G). All of the above results suggested that IL-8 promoted the progression of ICC.

### IL-8/CXCR2 promotes ICC by activating NF-κB

**FIGURE 2:**
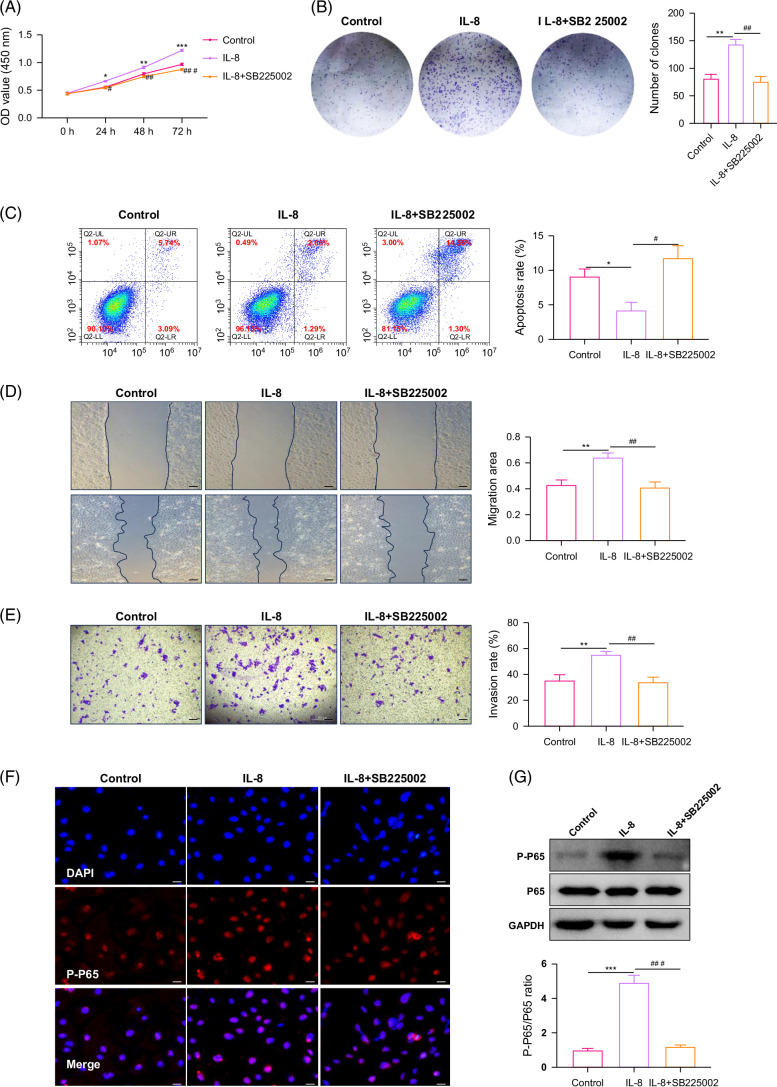
IL-8/CXCR2 promotes intrahepatic cholangiocarcinoma by activating NF-κB. (A) The proliferation ability of HuCCT1 cells with IL-8 treatment or SB225002 combined IL-8 treatment. (B) The clone formation ability of HuCCT1 cells with IL-8 treatment or SB225002 combined IL-8 treatment. (C) The apoptosis ratio of HuCCT1 cells with IL-8 treatment or SB225002 combined IL-8 treatment. (D) The migration ability of HuCCT1 cells with IL-8 treatment or SB225002 combined IL-8 treatment (scale bar=200 μm). (E) The invasion ability of HuCCT1 cells with IL-8 treatment or SB225002 combined IL-8 treatment (scale bar=200 μm). (F) The P-P65 fluorescence intensity of HuCCT1 cells with IL-8 treatment or SB225002 combined IL-8 treatment (scale bar=50 μm). (G) The expression of P-P65 protein with IL-8 treatment or SB225002 combined IL-8 treatment. Compared to the control group, *p<0.05, **p<0.01, and ***p<0.001. Compared to the IL-8 treatment group, #*p*<0.05, ##*p*<0.01, and ###*p*<0.001. Abbreviation: GAPDH, glyceraldehyde-3-phosphate dehydrogenase.

IL-8 generally exerts its biological effects by binding to receptors.^7^ It was found that CXCR2 was mainly expressed in ICC by detecting the levels of CXCR1 and CXCR2 in patients and cells (Supplemental Figures S2A and B, http://links.lww.com/HC9/B915). Together with the new findings of Meng et al, we speculated that IL-8 may act by binding to CXCR2 but not CXCR1. Next, ICC cells were treated with CXCR2 inhibitor (SB225002) combined with IL-8. It was found that CXCR2i inhibition suppressed the pro-tumor effect of IL-8, and even showed a certain inhibitory effect on tumors compared to the control group (Figures 2A–E). Our previous studies have shown that IL-8 can activate NF-κB, which plays an important role in the occurrence and development of ICC.^4^,^14^ We further examined the effect of SB225002 combined with IL-8 treatment on NF-κB. The results showed that IL-8-activated NF-κB was downregulated by SB225002 (Figures 2F and G). Substantially, NF-κB was involved in the ICC-promoting effect by IL-8/CXCR2.

### ALDH1A1 is highly expressed in cholangiocarcinoma and promotes ICC by activating NF-κB

**FIGURE 3:**
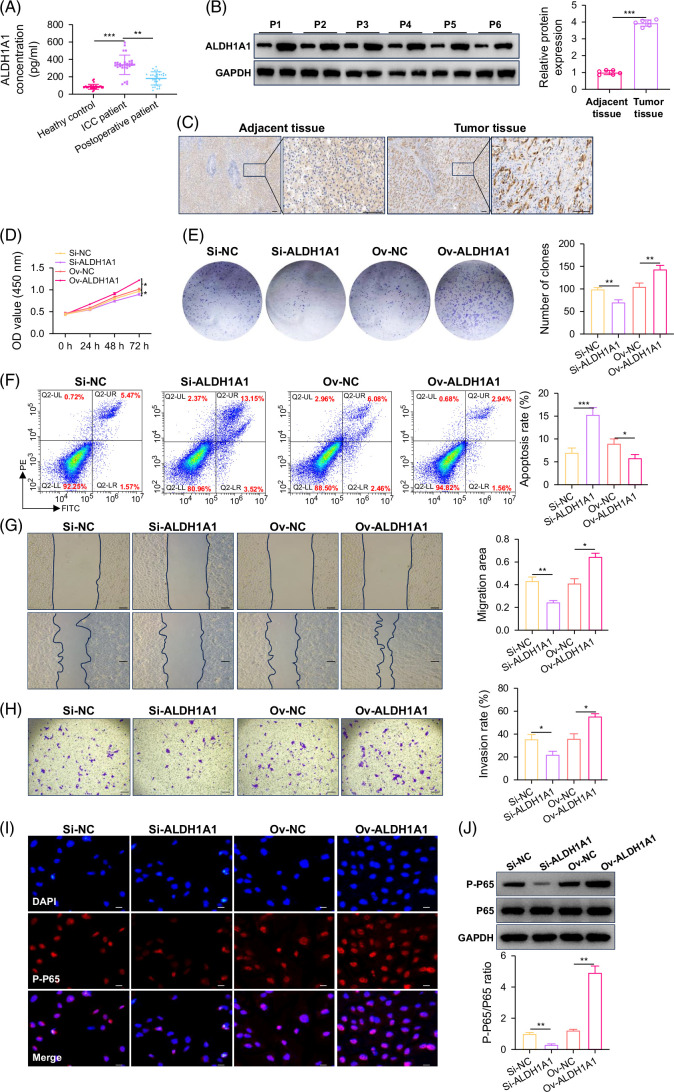
ALDH1A1 is highly expressed in ICC, and ALDH1A1 promotes ICC by activating NF-κB. (A) The level of ALDH1A1 concentration in 30 patients with ICC. (B) The expression of ALDH1A1 protein in ICC adjacent and tumor tissues. (C) The immunohistochemistry staining of ALDH1A1 in ICC adjacent and tumor tissues (scale bar=100 μm). (D) The proliferation ability of ICC cells with different ALDH1A1 expression. (E) The clone formation ability of ICC cells with different ALDH1A1 expression. (F) The apoptosis ratio of ICC cells with different ALDH1A1 expression. (G) The migration ability of ICC cells with different ALDH1A1 expression (scale bar=200 μm). (H) The invasion ability of ICC cells with different ALDH1A1 expression (scale bar=200 μm). (I) The P-P65 fluorescence intensity of ICC cells with different ALDH1A1 expression (scale bar=50 μm). (J) The expression of P-P65 protein of ICC cells with different ALDH1A1 expression. *p<0.05, ** *p*<0.01, and ***p<0.001. Abbreviations: GAPDH, glyceraldehyde-3-phosphate dehydrogenase; ICC, intrahepatic cholangiocarcinoma.

It was also found that patients with ICC had higher levels of ALDH1A1 expression compared to healthy controls by detecting the expression of ALDH1A1 in their serum. After surgical resection, the expression level of ALDH1A1 was significantly reduced compared to before surgery (Figure 3A). This finding was confirmed by western blot and IHC. The results showed that ALDH1A1 was upregulated in tumor tissues than in adjacent tissues (Figures 3B and C). Furthermore, we detected the expression level of ALDH1A1 in ICC cells and found that ICC cell lines had higher levels of ALDH1A1 expression compared to HIBEpiC cells (Supplemental Figures S3A–C, http://links.lww.com/HC9/B915). Next, HUCCT1 cells were chosen to transfect lentivirus, and the transfection efficiency was validated (Supplemental Figures S3D and E, http://links.lww.com/HC9/B915). After successfully constructing a cell line with differential expression of ALDH1A1, it was found that high expression of ALDH1A1 can significantly promote tumor cell colony formation, inhibit tumor cell apoptosis, promote tumor cell migration and invasion, and inhibit ALDH1A1 expression to exert an inhibitory effect on tumors (Figures 3D–H). These results suggested that ALDH1A1 promoted the progression of ICC. Our previous studies have shown that the promoting effect of ALDH1A1 on tumors is closely related to P65,^8^ and similar conclusions have also been found in ICC. Inhibiting ALDH1A1 can inhibit the activation of NF-κB (Figures 3I and J).

### IL-8 participates in promoting tumor progression by upregulating the expression of ALDH1A1 via activating NF-κB

**FIGURE 4:**
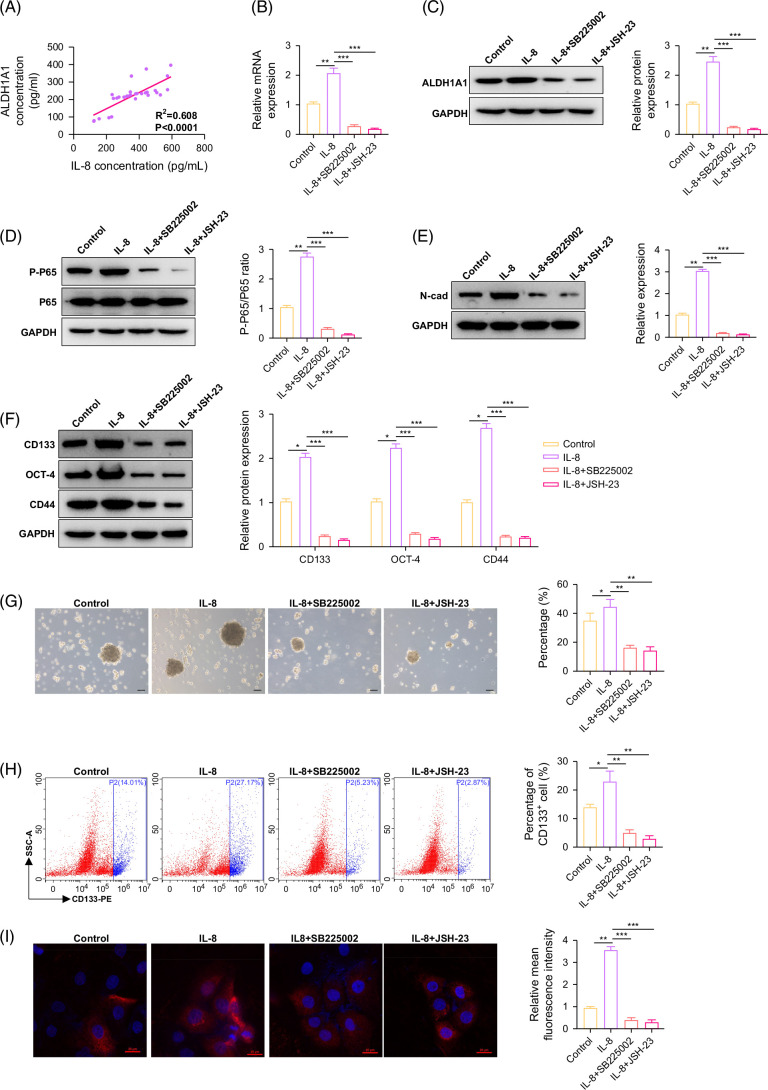
IL-8 participates in promoting tumor progression by upregulating the expression of ALDH1A1 via activating NF-κB. (A) The relationship of IL-8 and ALDH1A1 expression in 30 patients with intrahepatic cholangiocarcinoma. (B) The expression of ALDH1A1 mRNA by quantitative reverse transcription polymerase chain reaction. (C) The expression of ALDH1A1 protein by western blot. (D) The expression of P-P65 protein. (E) The expression of N-cadherin protein. (F) The expression of stem cell markers (CD133, OCT-4, CD44). (G) The tumorsphere formation ability of intrahepatic cholangiocarcinoma cells (scale bar=200 μm). (H) The expression of CD133 by flow cytometry. (I) The CD133 fluorescence intensity of intrahepatic cholangiocarcinoma cells (scale bar=20 μm). *p<0.05, ** *p*<0.01, and ***p<0.001. Abbreviations: GADPH, glyceraldehyde-3-phosphate dehydrogenase; N-cad, N-cadherin.

IL-8 and ALDH1A1 both play a promoting role in ICC from the above results. We further analyzed the correlation between IL-8 and ALDH1A1 and found a positive correlation in their expression in ICC (Figure 4A). We also analyzed the relationship between the expression level of plasma IL-8 and ALDH1A1 expression in tumor tissues and found that 14 cases in the IL-8 high expression group (N=15) were ALDH1A1 positive expression, while only 1 case in the IL-8 low expression group (N=15) was ALDH1A1 positive (*p*=0.014). These results indicated a significant positive correlation between IL-8 and ALDH1A1 expression. Then ICC cells were treated with IL-8 and found that IL-8 could upregulate the expression of ALDH1A1, while either SB225002 or NF-κB inhibitors (JSH-23) could inhibit ALDH1A1 expression upregulation by IL-8 (Figures 4B and C). Together, it was found that either SB225002 or JSH-23 could inhibit NF-κB activation by IL-8 (Figure 4D). It was found that IL-8 can regulate the expression level of N-cad, while either SB225002 or JSH-23 could inhibit this effect (Figure 4E). Further investigation revealed that IL-8 promotes tumor stemness in ICC, accompanied by upregulation of CD133, CD44, and OCT-4 expression, while either SB225002 or JSH-23 could inhibit the effect of IL-8 (Figures 4F–I). It can be seen that IL-8/CXCR2 can promote ICC by regulating the expression of ALDH1A1 through the NF-κB signaling pathway.

### ALDH1A1 promotes tumor progression by regulating IL-8 expression through activating NF-κB

We also found that ALDH1A1 can regulate the expression level of IL-8. Silencing ALDH1A1 could inhibit the expression of IL-8, while overexpression of ALDH1A1 could promote the expression of IL-8 (Figures 5A and B). Inhibition of NF-κB could downregulate the upregulated effect of ALDH1A1 on IL-8 (Figures 5C and D). Silencing ALDH1A1 expression could detriment the tumor-promoting effect of IL-8. While blocked IL-8 function by CXCR2i could detriment the tumor-promoting effect of ALDH1A1 (Figures 5E–N), accompanied by the changes in NF-κB (Figure 5O). This indicated that the expression of ALDH1A1 is crucial for IL-8 to promote ICC progression, and inhibiting IL-8/CXCR2 can also inhibit the tumor-promoting effect of ALDH1A1. The activation of NF-κB was involved in the regulation of ALDH1A1 by IL-8/CXCR2 and also was crucial for ALDH1A1 to regulate IL-8.

FIGURE 5ALDH1A1 promotes tumor progression by regulating IL-8 expression through activating NF-κB. (A) The expression of IL-8 mRNA in ICC cells with different ALDH1A1 expression. (B) The expression of IL-8 concentration in ICC cells with different ALDH1A1 expression. (C) The expression of IL-8 mRNA in ICC cells with JSH-23 treatment. (D) The expression of IL-8 concentration in ICC cells with JSH-23 treatment. (E) The proliferation ability of si-ALDH1A1 cells with IL-8 treatment. (F) The proliferation ability of si-ALDH1A1 cells with SB225002 treatment. (G, H) The effect of IL-8 treatment or SB225002 treatment on clone formation ability in different ALDH1A1 expression ICC cells. (I, J) The effect of IL-8 treatment or SB225002 treatment on apoptosis ratio in different ALDH1A1 expression ICC cells. (K, L) The effect of IL-8 treatment or SB225002 treatment on migration ability in different ALDH1A1 expression ICC cells (scale bar=200 μm). (M, N) The effect of IL-8 treatment or SB225002 treatment on invasion ability in different ALDH1A1 expression ICC cells (scale bar=200 μm). (O) The effect of IL-8 treatment or SB225002 treatment on expression of N-cadherin and P-P65 protein in different ALDH1A1 expression ICC cells. *p<0.05, **p<0.01, and ***p<0.001. Abbreviations: GADPH, glyceraldehyde-3-phosphate dehydrogenase; N-cad, N-cadherin.

**Figure:**
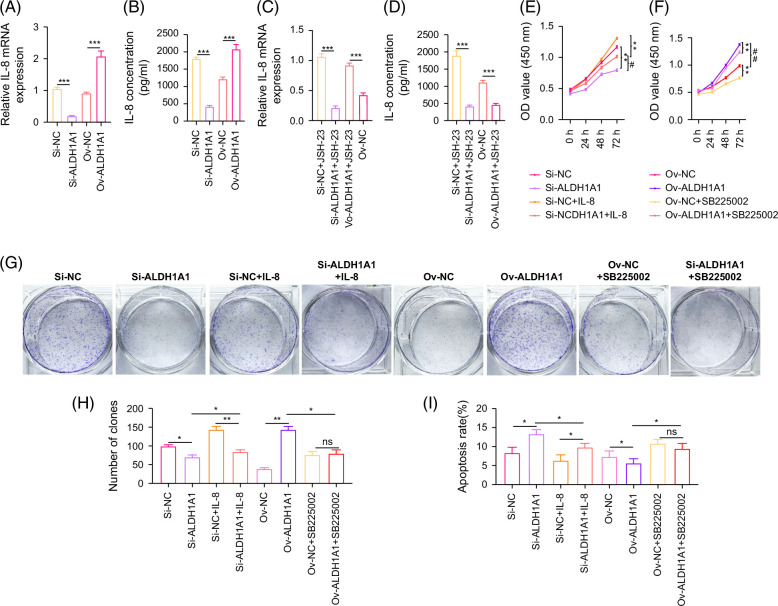

**Figure:**
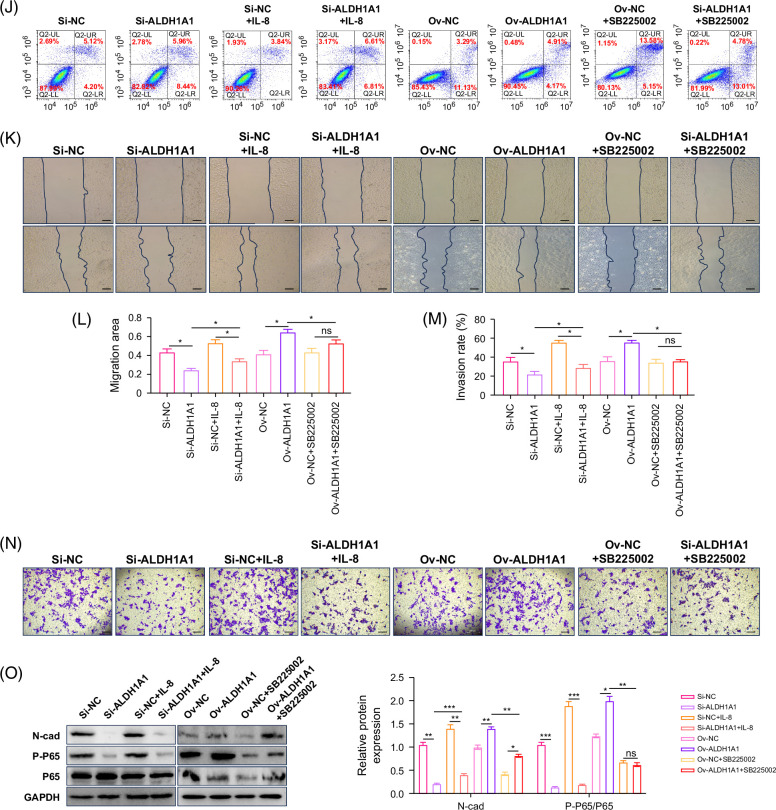

### Verification of the effect of IL-8/CXCR2–NF-κB–ALDH1A1 loop promotion on the progression of ICC in vivo

**FIGURE 6:**
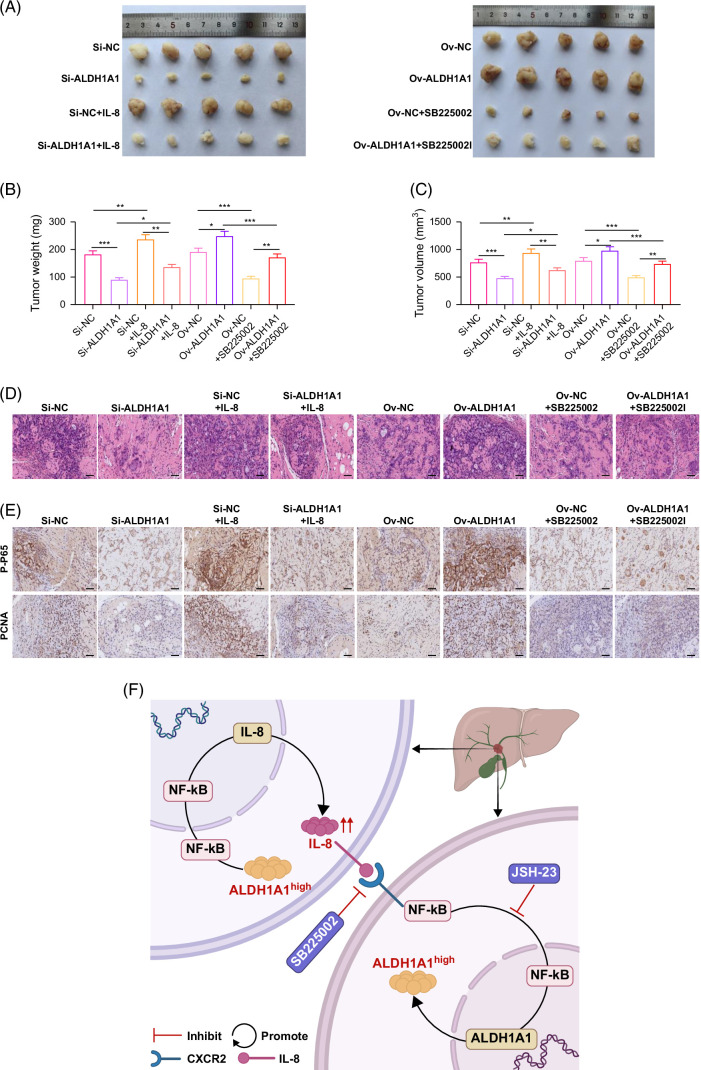
Verification of the effect of IL-8/CXCR2–NF-κB–ALDH1A1 loop promotion of the progression of ICC in vivo. (A) The effect of IL-8 treatment or SB225002 treatment on the tumor size in mice. (B) The effect of IL-8 treatment or SB225002 treatment on tumor weight in mice. (C) The effect of IL-8 treatment or SB225002 treatment on tumor volume in mice. (D) The hematoxylin and eosin staining of tumors (scale bar=100 μm). (E) The immunohistochemistry staining of P-P65 and PCNA in tumors (scale bar=100 μm). (F) Summary of IL-8/CXCR2–NF-κB–ALDH1A1 loop promotion the progression of ICC. *p<0.05, ** *p*<0.01, and ***p<0.001. Abbreviation: PCNA, proliferating cell nuclear antigen.

Subcutaneous tumors in nude mice were constructed. The above finding was confirmed by in vivo experiments. Silencing ALDH1A1 expression could detriment the tumor-promoting effect of IL-8. While blocked IL-8 function by CXCR2i could detriment the tumor-promoting effect of ALDH1A1 in vivo (Figures 6A–C). The IHC results showed that inhibiting ALDH1A1 or inhibiting IL-8/CXCR2 could suppress the expression of NF-κB and promote apoptosis of tumor cells (Figures 6D and E).

## DISCUSSION

This study clarified that IL-8-derived tumor cells could upregulate ALDH1A1 expression by activating the NF-κB signaling pathway, promoting tumor progression. Upregulation of ALDH1A1 could activate NF-κB to promote IL-8 secretion, forming a positive feedback loop to promote tumor invasiveness and cell stemness in ICC (Figure 6F). This provided new insights into the biological behavior of ICC and a scientific basis for further effective targeted therapy of ICC.

It was reported that CD133 and ALDH may be candidates for molecular markers of cholangiocarcinoma stem cells.^15^ Also, another study showed that TGF-β could promote epithelial–mesenchymal transition in ALDH1^positive^ cells, and IHC results of patients with cholangiocarcinoma confirmed that the prognosis of ALDH1^high^ group patients was worse.^16^ However, it was found that high expression of ALDH1A1 predicted better prognosis, while CD133 had no effect on prognosis in an early cholangiocarcinoma prognosis and recurrence prediction study. In this study, the stem cell marker panel (including CD133 and ALDH1A1) was evaluated on 178 cases of cholangiocarcinoma.^17^ Another study on the function of the ARID1A gene in ICC cells elucidated that individuals with ALDH1A1 positivity had a poorer prognosis, suggesting that ARID1A inhibition of ALDH1A1 expression could play a role in tumor suppression.^18^ Until now, the role of ALDH1A1 in ICC has been controversial. The reason for the differences may be that some previous studies included patients with hilar cholangiocarcinoma and extrahepatic cholangiocarcinoma, while some studies only focused on patients with ICC. Our research indicated that ALDH1A1 plays a tumor-promoting role in ICC. At the same time, a multi-omics analysis based on genomics, transcriptomics, proteomics, and phosphoproteomics was conducted on 102 samples of ICC patients, revealing an important precise molecular subtype in ICC-ALDH1^high^ subtype, and suggesting the combination of ALDH1A1 inhibitors in the treatment of such patients.^2^ This study further elucidated the tumor biological behavior of ALDH1A1 overexpression, including the positive feedback loop of IL-8 autocrine activation involving NF-κB.

It is well known that ALDH1A1 is a marker of stem cells. There was one study found that eugenol could enhance the inhibition of cisplatin on NF-κB signaling pathway and IL-8 secretion, thereby effectively reducing the proportion of ALDH1A1^high^ stem cells, reducing drug resistance, and increasing drug sensitivity in triple-negative breast cancer.^19^ Knockout of NF-κB expression using P65-specific small interfering RNA could significantly reduce the formation of CSCs and the secretion of IL-8, indicating that NF-κB/IL-8 is crucial for the formation of CSCs.^20^ It was found that the ERK5/NF-κB/IL-8 signaling axis can regulate tumor stem cell viability, and targeting this axis may eliminate tumor stem cell resistance in colon CSCs.^21^ And disrupting the inflammation-related CXCL8–CXCR1 signaling can inhibit the tumorigenicity of sporadic and colitis colon CSCs.^22^ Mesenchymal stem cells of gastric cancer could secrete IL-8 to induce the expression of PD-L1, and the expression and stem cell properties of PD-L1 can be reversed by blocking IL-8. Indicating that IL-8 is crucial for promoting and maintaining the stemness of tumor cells.^23^ These results were consistent with the findings of this study, which also indicated that IL-8 could upregulate the expression of ALDH1A1 and tumor stemness in ICC cells. This effect was also inhibited by SB225002 inhibition of CXCR2. At the same time, cells with upregulated ALDH1A1 expression could secrete more IL-8, and these processes were accompanied by the involvement of the NF-κB signaling pathway.

The role of IL-8 in ICC is attracting more and more attention. NF-κB activation and IL-8 upregulation played important roles in the pathogenic mechanisms of ICC caused by *Helicobacter pylori* and liver flukes.^24^,^25^ The study exploring the mechanism of ICC caused by primary sclerosing cholangitis revealed that NF-κB activation and increased IL-8 promoted the malignant transformation of cholangitis.^26^ Thymus phosphorylase also promoted tumor growth and angiogenesis by upregulating IL-8 expression.^27^ HOXB7 promoted invasion, metastasis, and poor prognosis of ICC, including the involvement of IL-8.^28^ CXCR2 small interfering RNA or inhibitor SB225002 to inhibit CXCR2, which could suppress the growth of ICC cells RBE and SSP25 and high expression of CXCR2 was also closely related to poor prognosis.^29^ This study also confirmed that CXCR2 was the main receptor that played a role in ICC, and when combined with IL-8, it activated NF-κB to promote the malignant biological behavior of ICC, including cell proliferation, invasion, and metastasis. At the same time, it also promoted the expression of ALDH1A1 and tumor stemness, while inhibiting CXCR2 or NF-κB could block the tumor promotion effect of IL-8.

In this study, the level of IL-8 in patients was measured just using the ELISA method. One study has clarified the overexpression of IL-8 in ICC tumor tissues and its relationship with prognosis.^7^ And our results were also similar to the conclusion of this study that IL-8 high expression was associated with lymphoma node metastasis. Meanwhile, it was reported that measurement of serum IL-8 may represent a good candidate to accurately estimate the number of tumor cells producing this chemokine in many cancers.^30^ The level of ALDH1A1 in patients was measured by ELISA, western blot, and IHC methods. All results supported the high expression of ALDH1A1 in tumors. However, the sample size of this study was relatively small. In future work, it is necessary to expand the sample size to verify the positive expression in tumor tissue and elevated levels in serum through various detection methods, as well as the consistency of expression in plasma and tissue, in order to make the results more reliable and provide a basis for the biomarker detection methods of ICC in the future.

In summary, this study clarified that IL-8-derived tumor cells could upregulate ALDH1A1 expression by activating the NF-κB signaling pathway, promoting tumor progression. Upregulation of ALDH1A1 could activate NF-κB to promote IL-8 secretion, forming a positive feedback loop to promote tumor invasiveness and cell stemness in ICC. This finding contributes to a deeper understanding of the biological behavior of ICC. Targeted inhibition of ALDH1A1, IL-8, or NF-κB signaling pathway activity can effectively suppress the proliferation, invasion, migration, and stemness of ICC, providing new insights for treatment.

## Supplementary Material

## Data Availability

The data that support the findings of this study are available from the corresponding author, Sulai Liu, upon reasonable request. Chuang Peng and Sulai Liu designed the research study. Yinghui Song, Jia Zhou, Yizhi Wang, Yueren Wang, Xinling Zhang, and Zexi Yin performed the research. Yufeng Li, Feicheng Yang, and Yuewei Tao collected and analyzed the data. Yinghui Song, Yufeng Li, and Yuewei Tao have been involved in drafting the manuscript and all authors have been involved in revising it critically for important intellectual content. This work was financially supported by the National Natural Science Foundation of China (No. 82303511), the Project of Hunan Provincial Health Commission (B202304017222), and the Hunan Provincial People’s Hospital RenShu fund (RS2022A07). The authors have no conflicts to report. Hunan Provincial People’s Hospital (the first affiliated hospital of Hunan Normal University) reviewed and approved the use of human tissue specimens and blood samples (No. 2024-108). Written informed consent for publication was obtained from all participants.
