# Supplementary material for: IL-8–NF-κB–ALDH1A1 loop promotes the progression of intrahepatic cholangiocarcinoma
Source: Hepatol Commun. 2025 Feb 26;9(3):e0664. doi: 10.1097/HC9.0000000000000664 (PMC11868433; doi:10.1097/HC9.0000000000000664)
Supplement: Supplementary file 1 [file hc9-9-e0664-s001.docx]

**Supplementary table 1 Primes for q-PCR**

| **Gene name** | **Forward primer** | **Reverse primer** |
| --- | --- | --- |
| *IL-8* | 5’-TTGGCAGCCTTCCTGATTT-3’ | 5’-TCAAAAACTTCTCCACAACCC-3’ |
| *ALDH1A1* | 5'-TGTTAGCTGATGCCGACTTG-3' | 5'-TTC TTAGCCCGCTCAACACT-3' |
| *GAPDH* | 5‘-AATCCCATCACCATCTTCCA-3’ | 5‘-CCTGCTTCACCACCTTCTTG-3’ |

**Supplementary table 2 The antibodies information**

| **Antibody** | **Manufacturer** | **Item No.** | **Dilution** |
| --- | --- | --- | --- |
| ALDH1A1 for WB | CST | #54135 | 1:1000 |
| P-P65 for WB | CST | #3033 | 1:1000 |
| P65 for WB | CST | #8242 | 1:1000 |
| CXCR1 for WB | Zenbio | 160229 | 1:1000 |
| CXCR2 for WB | Zenbio | 251676 | 1:1000 |
| N-Cadherin for WB | CST | #13116 | 1:1000 |
| CD133 for WB | CST | #64326 | 1:1000 |
| OCT-4 for WB | CST | #2750 | 1:1000 |
| CD44 for WB | CST | #37259 | 1:1000 |
| GAPDH for WB | CST | #2118 | 1:2000 |
| ALDH1A1 for IHC | CST | #54135 | 1:400 |
| P-P65 for IHC | Zenbio | 340830 | 1:200 |
| PCNA for IHC | Zenbio | 200947-2E1 | 1:200 |
| P-P65 for IF | CST | #3033 | 1:800 |
| CD133 for IF | CST | #64326 | 1:400 |
| CD133 for FCM | CST | #64326 | 1:500 |
| Goat anti-Rabbit IgG Antibody (H+L), PE Conjugated | Bioss | Bs-0295G | 1:100 |
| Anti-rabbit IgG, HRP-linked Antibody | CST | #64326 | 1:2000 |


**Supplementary table 3 Relationship of IL-8 expression with clinicopathological factors in 30 ICC patients**

| **Clinical feature** | **Characteristics** | **IL-8 expression (N)** | | **P** |
| --- | --- | --- | --- | --- |
|  |  | **Low** | **High** |  |
| Age (years) | ≤60 | 10 | 8 | 0.7104 |
|  | >60 | 5 | 7 |  |
| Sex | Female | 9 | 10 | 0.999 |
|  | Male | 6 | 5 |  |
| CEA | ≤5 | 8 | 6 | 0.7152 |
|  | >5 | 7 | 9 |  |
| Viral hepatitis | No | 9 | 5 | 0.2723 |
|  | Yes | 6 | 10 |  |
| Stones | No | 12 | 8 | 0.2451 |
|  | Yes | 3 | 7 |  |
| Lymph node metastasis | No | 12 | 4 | **0.0092** |
|  | Yes | 3 | 11 |  |
| ALDH1A1 | Negative | 8 | 1 | **0.0142** |
|  | Positive | 7 | 14 |  |
