# Supplementary material for: IL-8–NF-κB–ALDH1A1 loop promotes the progression of intrahepatic cholangiocarcinoma
Source: Hepatol Commun. 2025 Feb 26;9(3):e0664. doi: 10.1097/HC9.0000000000000664 (PMC11868433; doi:10.1097/HC9.0000000000000664)

SFigure 1 IL-8 is overexpressed in tumors and tumor cells. (A) The level of CXCL8 expression in different tumor types from using the TCGA database. (B) The level of IL-8 mRNA expression in different tumor cell lines. (C) The level of IL-8 concentration in different tumor cell lines.


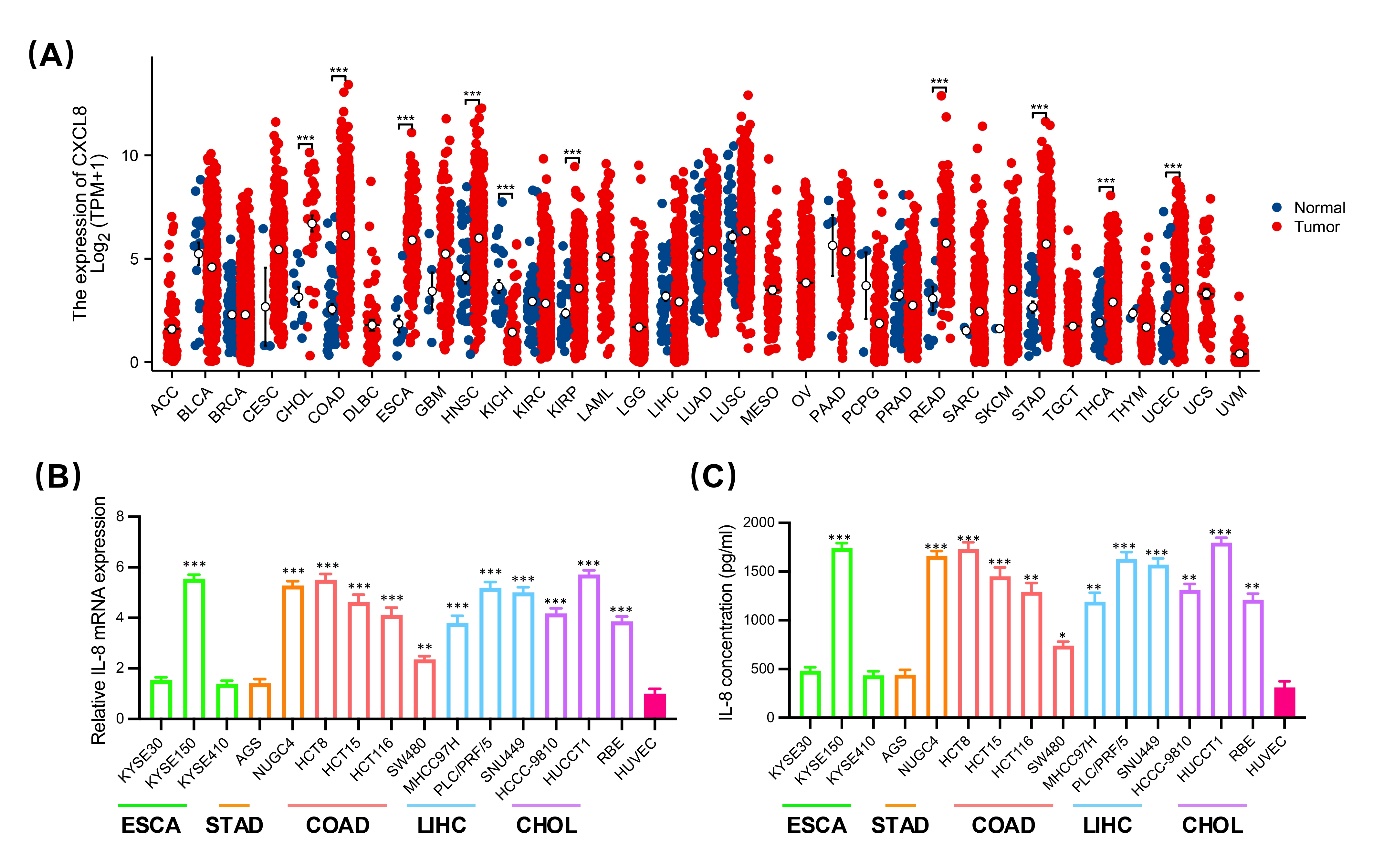


SFigure 2 CXCR2 but not CXCR1 is mainly expressed in ICC. (A) The expression of CXCR1 and CXCR2 protein in ICC patients. (B) The expression of CXCR1 and CXCR2 protein in ICC cell lines.


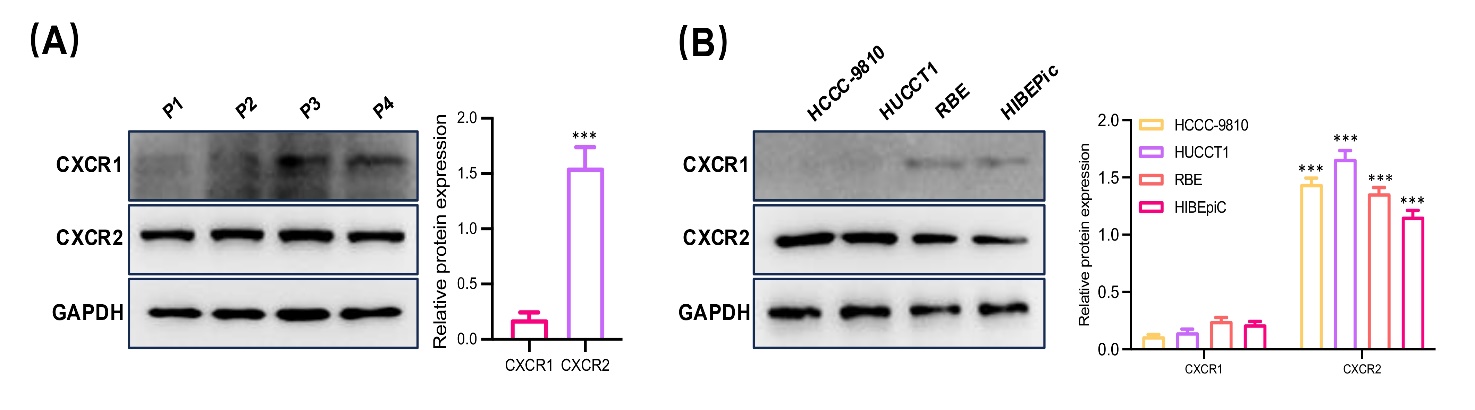


SFigure 3 ALDH1A1 is overexpressed in ICC cell lines. (A) The expression of ALDH1A1 mRNA in ICC cell lines. (B) The level of ALDH1A1 concentration in ICC cell lines. (C) The expression of ALDH1A1 protein in ICC cell lines. (D) The expression of ALDH1A1 mRNA in transfection cells. (E) The expression of ALDH1A1 protein in transfection cells. *P<0.05, ** P<0.01, ***P<0.001.


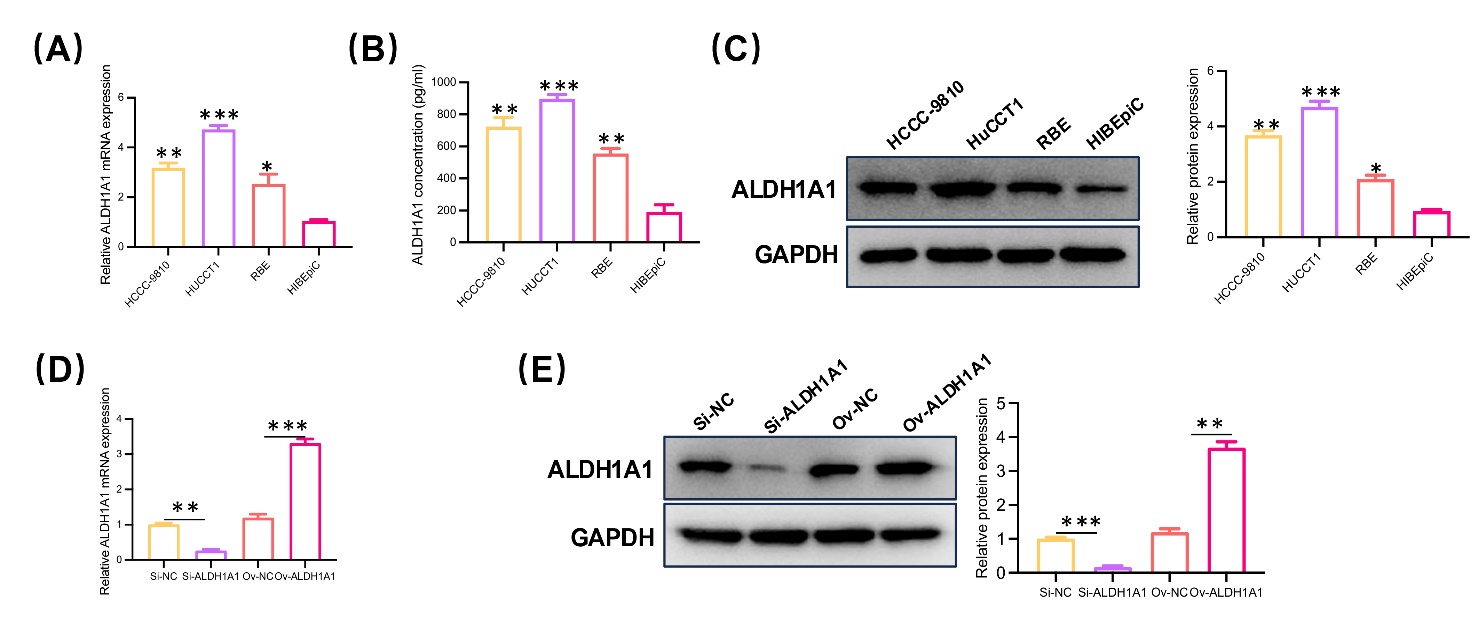

Supplement: Supplementary file 2 [file hc9-9-e0664-s002.docx]
